# Comparative Aspects of Molecular Mechanisms of Drug Resistance through ABC Transporters and Other Related Molecules in Canine Lymphoma

**DOI:** 10.3390/vetsci2030185

**Published:** 2015-08-12

**Authors:** Hirotaka Tomiyasu, Hajime Tsujimoto

**Affiliations:** 1 Department of Veterinary Clinical Sciences, College of Veterinary Medicine, University of Minnesota, 1352 Boyd Ave, St. Paul, MN 55108, USA; 2 Masonic Cancer Center, University of Minnesota, Minneapolis, 420 Delaware Street SE, Minneapolis, MN 55455, USA; 3 Department of Veterinary Internal Medicine, Graduate School of Agricultural and Life Sciences, The University of Tokyo, 1-1-1 Yayoi, Bunkyo-ku, Tokyo 113-8657, Japan; E-Mail: atsuji@mail.ecc.u-tokyo.ac.jp

**Keywords:** ABC transporters, dog, epigenetics, LRP, MAPK pathways

## Abstract

The most important causes of treatment failure in canine lymphoma include intrinsic or acquired drug resistance. Thus, elucidation of molecular mechanisms of drug resistance is essential for the establishment of better treatment alternatives for lymphoma patients. The overexpression of drug transporters is one of the most intensively studied mechanisms of drug resistance in many tumors. In canine lymphoma, it has also been shown that the overexpression of drug efflux pumps such as *P*-glycoprotein is associated with drug-resistant phenotypes. Canine lymphoma has many pathological similarities to human non-Hodgkin’s lymphoma, and they also share similar molecular mechanisms of drug resistance. We have previously demonstrated the association of the overexpression of drug transporters with drug resistance and indicated some molecular mechanisms of the regulation of these transporters’ expressions in canine and human lymphoid tumor cells. However, it has also been indicated that other known or novel drug resistance factors should be explored to overcome drug resistance in lymphoma. In this review, we summarize the recent findings on the molecular mechanisms of drug resistance and possible strategies to develop better treatment modalities for canine lymphoma from the comparative aspects with human lymphoid tumors.

## 1. Introduction

Lymphoma is the most common hematologic neoplasm in dogs [1], and it is a representative neoplasm that responds to conventional chemotherapy such as CHOP, which comprises cyclophosphamide, doxorubicin, vincristine and prednisolone. It has been reported that treatment with chemotherapeutic agents for dogs with lymphoma results in high rates of complete response, ranging from 76.3% to 92.3% [2,3]. However, most canine patients with lymphoma undergo relapses, and efficacy of rescue protocols is limited owing to the development of resistance to the agents used in remission-induction therapy. Based on these backgrounds, the molecular mechanisms of the drug resistance in lymphoma cells have been intensively investigated for the development of more effective treatments for canine lymphoma, and most studies have focused on the overexpression of drug transporters such as P-glycoprotein (P-gp). However, overexpression of P-gp has been demonstrated in only a small proportion of multidrug-resistant canine lymphoma cases.

**Figure 1:**
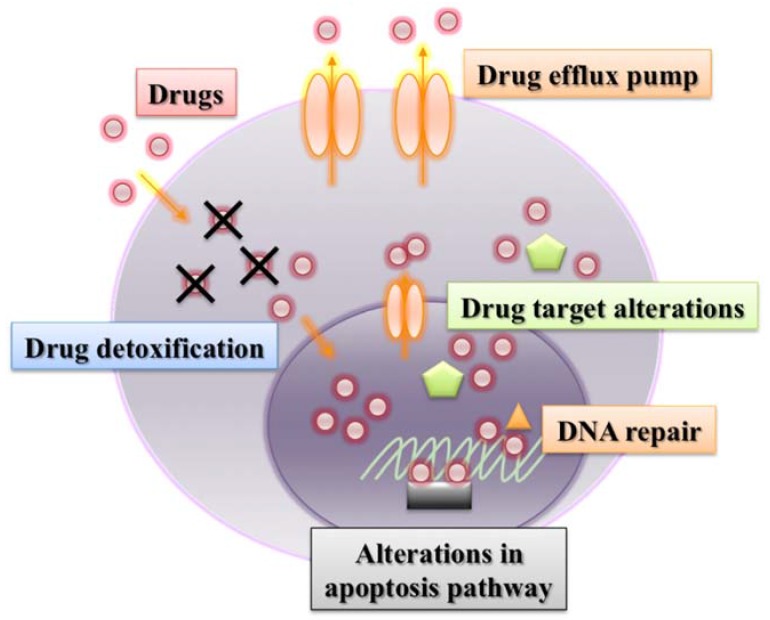
Representative molecular mechanisms of drug resistance.

Canine tumors are thought to be a spontaneous animal model system of human cancer biology [4]. In particular, canine lymphoma shares many similarities with human non-Hodgkin’s lymphoma (NHL) [5]. The standard treatment for human NHL also includes CHOP-based chemotherapy, and the addition of rituximab, which is a humanized monoclonal antibody that binds to the CD20 antigen, to the CHOP protocol (R-CHOP), which has provided major improvement in the therapy for human NHL of B-cell origin [6]. However, a substantial population of human patients with NHL has innate drug resistance or undergoes relapse after achieving response to chemotherapy. Development of drug resistance in tumor cells is another major obstacle in the treatment for human NHL patients [7]. In human medicine, several molecular mechanisms of drug resistance have been revealed in many tumors (Figure 1). As many similarities have been observed in the molecular mechanisms of drug resistance between canine and human tumor cells, it is important to investigate the mechanisms from the aspects of comparative oncology. Here, we summarize the recent findings on the molecular mechanisms of drug resistance and possible strategies to develop better treatment modalities for canine lymphoma from the comparative aspects with human medicine.

## 2. Transporters Associated with Drug Resistance

### 2.1. P-gp

ATP-binding cassette (ABC) transporters are a family of drug efflux pumps, and 49 members of this family have been identified in human medicine [8]. They are divided into seven subfamilies, ABCA through ABCG. These transporters are expressed on the cellular membrane and transport their substrates across the membrane in an ATP-dependent manner. P-gp, which is coded by the *ABCB1* gene, is one of the ABC transporters. The overexpression of P-gp reduces the intracellular concentration of the substrate agents, including vincristine and doxorubicin, commonly used in the treatments for lymphoma patients (Table 1) [8]. Among molecules that induce a drug resistance phenotype, P-gp is one of the most intensively studied molecules in humans [9] and dogs [10]. In human medicine, NHL cells have been found to express P-gp [11]. The roles of P-gp in the development of drug-resistant phenotypes are indicated in NHL patients since P-gp inhibitors have been shown to be effective in patients harboring a drug-resistant phenotype [12,13,14]. The associations of P-gp expression with drug resistance have also been shown in canine lymphoma. The transduction of the canine *ABCB1* gene has been reported to induce resistance to several chemotherapeutic agents in the canine cell line [15]. Furthermore, an association between P-gp expression and the drug-resistant phenotype has been reported in dogs with lymphoma [16,17,18]. The rates of dogs that expressed P-gp were high at relapse or acquisition of drug-resistant phenotypes [17,18], and canine lymphoma patients with the expression of P-gp before chemotherapy had low response rate and short survival time [16]. We previously showed the high expression level of the *ABCB1* gene in a proportion of dogs with lymphoma that developed multidrug resistance [19]. These observations suggested that the overexpression of P-gp is associated with drug-resistant phenotypes in at least some canine lymphoma patients.

### 2.2. Other ABC Transporters

Other representative ABC transporters associated with drug resistance in human tumors include multidrug resistance-associated protein 1 (MRP1) and breast cancer resistance protein (BCRP) [8].

MRP1, coded by the *ABCC1* gene, has been shown to be associated with resistance to chemotherapeutic agents including vincristine and doxorubicin in several human tumors (Table 1) [8]. Although the studies on the clinical implications of the expression of MRP1 in human NHL are limited, Ohsawa *et al.* revealed that the complete remission rate of the patients who expressed MRP1 was significantly lower than that of the patients without the expression of this transporter [20]. In veterinary medicine, it was shown that MRP1 was responsible for vinblastine and cisplatin resistance in canine mammary tumor cell lines [21], and the expression of the *ABCC1* gene was generally detected in all samples of the 103 primary canine mammary tumors [22]. We have previously shown that there is no significant difference in the expression level of *ABCC1* mRNA between canine lymphoma patients with and without a drug-resistant phenotype [19]. A recent study has shown a low transporter activity of MRP1 in canine lymphoma samples before chemotherapy [23]. Thus, the association of MRP1 expression with drug resistance is still unclear in canine lymphoma.

BCRP, coded by the *ABCG2* gene, has also been shown to induce resistance to various anticancer drugs (Table 1) [8]. In human NHL patients, increased expression of the *ABCG2* gene or BCRP has been shown to be associated with poor prognosis [24,25]. In veterinary medicine, the association of the expression of BCRP with resistance to doxorubicin, cyclophosphamide and cisplatin was reported in canine mammary tumor cell lines [21,22]. As to canine lymphoma, our previous study showed no significant difference in the *ABCG2* gene expression level between canine lymphoma patients with and without drug resistance [19]. However, Zandvliet *et al.* revealed that *ABCG2* gene upregulation was associated with the drug-resistant phenotype in canine T-cell lymphoma patients [26].

**Table 1:** Representative conventional chemotherapeutic agents that are substrates of drug transporters.

P-gp	MRP1	BCRP	LRP
*Vinca* alkaloids	*Vinca* alkaloids	Anthracyclines	*Vinca* alkaloids
Anthracyclines	Anthracyclines	Nucleoside analogs	Anthracyclines
Epipodophyllotoxins	Epipodophyllotoxins	Epipodophyllotoxins	Epipodophyllotoxins
Taxanes	Camptothecin	Camptothecin	Taxanes
Camptothecin	Methotrexate	Methotrexate	Platinum-containing drugs
Methotrexate			Nitrogen mustard
Other antibiotics (Actinomycin-D, Mitomycin-C)			
		

### 2.3. Lung Resistance Protein (LRP)

Lung resistance protein (LRP), which is identical to major vault protein, was first identified in lung cancer cell lines with drug resistance not associated with P-gp [27]. It has a different function from ABC-transporters and redistributes drugs from the nucleus to the cytoplasm, as it is located primarily in the cytosol and nuclear membrane [28]. LRP has also been shown to be associated with resistance to several anticancer drugs (Table 1) [29,30]. In human NHL, the association of LRP expression with resistance against doxorubicin was indicated [31,32], and LRP expression-positive NHL patients had poorer outcomes compared to those without its expression [33]. However, there are few studies on the roles of LRP in drug resistance in veterinary medicine. An immunohistochemical study showed LRP expression in 85% of canine primary pulmonary carcinomas patients [34]. We previously examined the mRNA expression of the *LRP* gene in dogs with lymphoma, but there was no significant difference between dogs with and without drug resistance [19]. Therefore, the clinical implications of LRP expression remain to be clarified in canine lymphoma cases.

## 3. Molecular Mechanisms of Regulation of the Expression and Enhancement of P-gp Functions

The responsible molecular mechanisms for the overexpression or enhancement of functions of drug transporters have been investigated mainly for P-gp and the ABCB1 gene in both human and canine tumors. The representative molecular mechanisms include genetic changes such as amplification of a gene, mutations and chromosomal rearrangements, and transcriptional or posttranscriptional regulations by alternative promoters, epigenetic regulations and intracellular signal pathways.

### 3.1. Genetic Changes

One of the genetic changes that induces the overexpression of genes is gene amplification. In some previous studies in human medicine [35,36], it was shown that amplification of the *ABCB1* gene was induced in several tumor cells by stepwise exposure to some chemotherapeutic agents, which is the conventional method to establish drug-resistant cell lines. In one study, amplification of the *ABCB1* gene was observed after exposures to doxorubicin, colchicine, vinblastine or vincristine in epidermoid carcinoma, colon carcinoma, breast carcinoma and lymphoma cell lines [36]. However, there has been no study on the amplification of the *ABCB1* gene in canine tumor cells with drug resistance. 

In human and Chinese hamster tumor cell lines, some mutations of the *ABCB1* gene have been reported to be associated with drug resistance [37,38,39]. The mutations at the valine residue at position 185 of human P-gp resulted in increased resistance against vinblastine or colchicine [37], and the substitutions of the amino acids within the sixth transmembrane domain enhanced the resistance against actinomycin D [38]. In addition, it is known in human medicine that several mutations in the *ABCG2* gene, such as those at position 482, lead to changes in substrate preferences and resistance to several drugs including doxorubicin [40,41,42,43]. However, the associations of the mutations of the *ABCB1* or *ABCG2* gene with drug resistance are unclear in human and canine lymphomas.

Other genetic changes that induce the overexpression of drug transporters include chromosomal translocations. In a previous study, the translocations resulting in hybrid mRNA containing *ABCB1* gene mRNA were observed with or without co-amplifications of these hybrid genes in some drug-resistant human tumor cell lines and in patients with refractory leukemia [39]. However, there has been no study on the development of chromosomal translocation involving the *ABCB1* gene during the development of drug resistance in human or canine lymphoma cells.

### 3.2. Alternative Promoter

**Figure 2:**
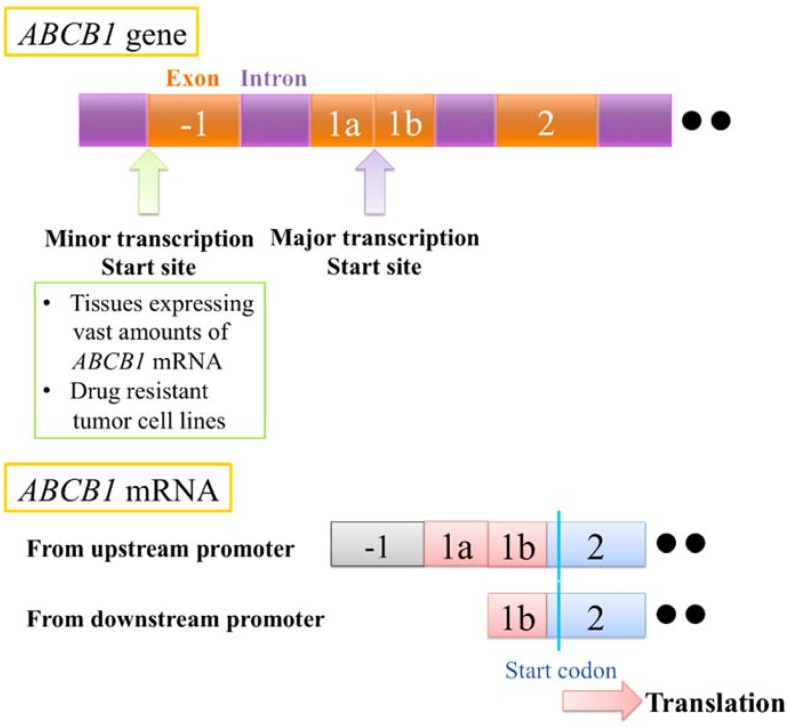
Alternative promoter of human ATP-binding cassette (*ABC)B1* gene.

It is known that the human *ABCB1* gene has two major promoter regions, “upstream” and “downstream” [44,45,46,47,48] (Figure 2). The “downstream” promoter is normally used, but the minor “upstream” promoter is used in tissues expressing vast amounts of *ABCB1* mRNA (such as the adrenal grand, liver and colon) or tumor cells overexpressing the *ABCB1* gene [46]. In the drug-resistant tumor cell lines that were established from stepwise exposure to several drugs, *ABCB1* transcripts from only upstream or both upstream and downstream were observed, along with increases in the amounts of the transcripts [45,46,48]. Huff *et al.* also revealed that the expression of transcripts derived from the upstream promoter were regulated by the genomic sequencing that includes a human endogenous retroviral long-terminal repeat in tumor cell lines including lymphoma cells [36]. The presence of the alternative promoter was also suspected in the canine *ABCB1* gene [49]. In this study, the suspected “downstream” promoter in intron 1 was shown to contain regulatory elements such as AP-1, AP-2 and SP-1. However, the association of this suspected promoter with overexpression of the *ABCB1* gene or the drug-resistant phenotype has not been reported.

### 3.3. Epigenetic Regulations

The changes in epigenetic regulations including DNA methylation, histone modifications and miRNA have recently been focused upon as the mechanisms that induce overexpression of the *ABCB1* gene.

DNA hypermethylation within the CpG island in the promoter regions is generally associated with the transcriptional silencing of the associated gene [50]. In human medicine, an inverse correlation has been observed between DNA methylation rates in the promoter region and *ABCB1* mRNA expression levels in T-cell leukemia [51,52], breast cancer [53,54] and other tumor cell lines [55,56]. Similar negative correlations have been observed in primary tumor cells from human patients with acute myeloid leukemia [57] or bladder cancer [58]. In terms of lymphoma, a study examined the methylation status of the *ABCB1* gene in patients with hematopoietic malignancies, including five NHL patients, but the detailed methylation status of the gene in those NHL patients was not described [59].

Histone modifications such as methylation, acetylation and phosphorylation are also known to regulate the expression of the associated gene. Among them, histone acetylation is almost invariably associated with the activation of transcription [60]. Baker *et al.* showed the regulation of *ABCB1* mRNA expression by histone H3 acetylation using a human acute T-cell leukemia cell line [61].

In veterinary medicine, we previously examined the epigenetic regulations of the *ABCB1* gene in canine lymphoid tumor cell lines [62]. In this study, we revealed that DNA methylation and histone acetylation within the promoter region of the *ABCB1* gene were associated with the regulations of its expression in canine lymphoid tumor cell lines. We also examined the DNA methylation status in the promoter regions of the *ABCB1* gene in 27 canine B-cell lymphoma patients [63], but the CpG island of this gene was hypomethylated in most dogs in both the chemotherapy-sensitive and -resistant patient groups, which meant that the DNA methylation status might not change during treatment in dogs with B-cell lymphoma.

Other regulation systems of gene expression include miRNAs. miRNAs are single-strand non-coding RNAs that mostly bind to the 3′-untranslated region (UTR) of the targeted mRNA, although binding to the coding region or the 5′-UTR may also occur [64]. miRNAs can regulate the expression of targeted mRNAs post-transcriptionally, and full complementary binding leads to mRNA cleavage while partial complementary binding leads to translational repression [65,66,67]. In human medicine, miR-451, miR-331-5p, miR-27a, miR-298 and miR-145 were shown to regulate the expression of the *ABCB1* gene by direct interaction with 3′-UTR [68,69,70,71]. In addition, miR-451, miR-27a, miR-21, miR-130a, miR-let-7, miR-137, miR-200c, miR-122, miR-138 and miR-10a/b were suggested to regulate *ABCB1* gene expression indirectly by targeting other mRNAs that code the proteins associated with the activation of *ABCB1* gene expression [72,73,74,75,76,77,78,79]. In human acute lymphoblastic leukemia (ALL) cell lines, miR-455-3p was shown to be related to P-gp expression level [80]. However, there has been no study on the regulations of *ABCB1* gene or P-gp expression by miRNAs in veterinary medicine.

### 3.4. Activation of Intracellular Signaling

The accumulating evidence indicates that certain signaling pathways are involved in the regulation of *ABCB1* gene and P-gp expression.

Mitogen Activated Protein Kinase (MAPK) signaling pathways are the representative pathways involved in the regulation of *ABCB1* gene and P-gp expression. MAPK pathways include three pathways: the extracellular signal-regulated kinase (ERK) pathway, the p38 MAPK pathway and the c-Jun N-terminal kinase (JNK) pathway. 

The activation of the MAPK/ERK pathway was associated with the upregulation of P-gp and the *ABCB1* gene in several human tumor cells [81,82,83,84], which indicated that the MAPK/ERK pathway regulates *ABCB1*/P-gp expression at both the transcriptional and posttranscriptional levels. Also in the human B-cell lymphoma cell line, the activation of the MAPK/ERK pathway was shown to upregulate *ABCB1* gene expression through increased expression or nuclear translocation and DNA-binding activity of YB-1, the transcription factor that regulates *ABCB1* gene expression [82]. We also previously revealed the upregulation of *ABCB1* gene expression and downregulation of the *ABCG2* gene through the activation of the MAPK/ERK pathway in human T- and B-ALL cell lines [85].

The association of the activation of the p38 MAPK pathway with *ABCB1* gene expression has been controversial. A previous study showed that the activation of the p38 MAPK pathway decreased the expression of the *ABCB1* gene in human hepatocellular carcinoma cell lines [86], whereas inhibition of this pathway decreased the *ABCB1* gene expression in human gastric cancer cell lines [87]. Another study showed that the inhibition of the p38 MAPK pathway did not change the expression level of P-gp in human colorectal cancer cell lines [81]. 

The JNK pathway is also known to be involved in the downregulation of *ABCB1* gene expression in several human tumor cells. The activation of the JNK pathway resulted in decreased *ABCB1* gene expression through the binding of c-Jun, the transcription factor in the downstream of JNK pathway, to the promoter region of the *ABCB1* gene in human ovarian cancer cell lines [88] and lung cancer cell lines [89]. Nuclear factor kappa B (NF-κB) also directly binds to the promoter region of the *ABCB1* gene and upregulates its expression [89,90], and the JNK pathway has been shown to inhibit NF-κB [89]. We also previously revealed that the activation of the JNK pathway upregulated *ABCG2* gene expression in human ALL cell lines [85].

Other intracellular signaling pathways that are associated with the regulation of *ABCB1* gene expression include the phosphatidylinositol 3-OH kinase (PI3K)-Akt pathway [91,92,93] and Wnt/β-catenin [94,95,96].

In veterinary medicine, we revealed the upregulation of *ABCB1* and *LRP* gene expression through the activation of the MAPK/ERK pathway by examining the side population, which is thought to contain the cancer stem cell fraction, in canine lymphoma cell lines [97]. Our previous studies also revealed that the JNK pathway downregulated *ABCB1* gene expression [98] and the MAPK/ERK and JNK pathways downregulated *ABCG2* gene expression [99] in those lymphoma cell lines.

## 4. Possible Strategies to Overcome Drug Resistance through Overexpression of Drug Transporters 

Since many studies indicated the association of the overexpression of drug transporters with drug-resistant phenotypes, a number of strategies have been proposed to overcome the drug-resistant phenotypes. We summarize these strategies here, focusing on those involving inhibition of the function and reduction of the expression of P-gp.

### 4.1. Inhibition of the P-gp Functions

The agents for the inhibition of P-gp functions are generally classified into three generations [100]. The first-generation P-gp inhibitors includes verapamil, quinine and cyclosporine; the second-generation includes valspodar (PSC-833) and biricodar (VX-710); and the third includes elacridar (GF-120918), laniquidar (R101933), zosuquidar (LY335979) and tariquidar (XR9576). All three generations were shown to inhibit P-gp function in many tumor cells *in vitro*, including lymphoid tumors, and some adverse effects observed with the first- and second-generation agents, such as the inhibition of cytochrome P450 3A, were decreased in the third-generation inhibitors [101]. However, a number of clinical trials have failed to show the benefits of using these inhibitors in solid and hematopoietic tumors in human medicine due to the severe toxicity or the limited clinical benefits [102]. These P-gp inhibitors were also used to reverse the drug-resistant phenotype of canine tumor cells *in vitro*. We previously showed that cyclosporine A could reestablish sensitivity to vincristine in canine drug-resistant lymphoma cells [62]. Zandvliet *et al.* also revealed that valspodar could reverse the resistance to doxorubicin and vincristine in a canine lymphoid cell line [103]. However, there no clinical trial is reported that has examined the effects of these inhibitors in veterinary medicine.

Recently, it has been shown that some tyrosine kinase inhibitors (TKIs) also inhibit the functions of P-gp and BCRP. The representative TKIs that modulate the functions of P-gp or BCRP include imatinib, dasatinib and nilotinib (BCR-ABL kinase inhibitors); gefitinib, erlotinib and lapatinib (epidermal growth factor receptor [EGFR] kinase inhibitiors); and sunitinib (an inhibitor that targets multiple kinases such as platelet-derived growth factor receptor [PDGFR] and vascular endothelial growth factor [VEGFR]) [104]. These TKIs are the substrates or modulators of P-gp or BCRP, which means that they have the potential to alter the pharmacokinetics and toxicity of chemotherapeutic drugs that are substrates of these transporters. Actually, it has been reported in previous studies that the addition of these TKIs enhances the sensitivity to transporter substrates, such as vincristine, in several human tumors including leukemic cells [104]. In veterinary medicine, one *in vitro* study has also shown that masitinib, which inhibits c-Kit, PDGFR and fibroblast growth factor receptor (FGFR), has antiproliferative effects, inhibits the drug efflux function of P-gp and increases the sensitivity to doxorubicin in a drug-resistant canine lymphoid tumor cell line [105]. Further studies are needed to examine the benefits of the addition of TKIs to chemotherapy for canine lymphoma in clinical trials.

### 4.2. Reduction of P-gp Expression

Alternative approaches to overcome drug resistance due to the overexpression of P-gp include the inhibition of *ABCB1* gene or P-gp expression. Many studies have shown that the expression levels of *ABCB1* mRNA or P-gp could be decreased *in vitro* by using molecular biology techniques such as antisense oligonucleotides, hammerhead ribozymes and short-interfering RNA (siRNA) in human medicine [102]. However, the sufficient decrease of P-gp expression has not been obtained and the safe delivery of the constructs has not been established *in vivo* [106,107].

Based on the observations that some intracellular signaling pathways are involved in the regulation of *ABCB1* gene expression, some studies have tried to decrease the expression of this gene by modulating the activations of intracellular signaling pathways using drugs such as TKIs. A previous study has shown that ponatinib, which is the inhibitor of BCR-ABL and fms-like tyrosine kinase 3 (FLT3), is a substrate of P-gp and can reduce the amounts of *ABCB1* mRNA in human chronic myelogenous leukemia cell line [108]. In human T-acute leukemia cell lines, it was reported that the Akt inhibitor, perifosine, induced the downregulation of P-gp and caspase-dependent apoptosis through the activation of the JNK pathway [109]. Based on these findings, we examined the effects of perifosine against canine lymphoid tumor cell lines. We revealed that this drug also had antitumor effects in canine lymphoid tumor cells and decreased *ABCB1* gene expression through activation of the JNK pathway, leading to a reduction of IC_50_ values for vincristine in a canine lymphoma cell line [98].

## 5. Other Mechanisms Associated with Drug Resistance

### 5.1. Drug Target Alterations

In human medicine, molecular targeted therapy has been established in many tumors where the specific molecules involved in cancer cell growth and survival are targeted by drugs such as antibodies or TKIs. The major causes of drug resistance in these therapeutic strategies include alterations in the targeted molecules. The representative molecular targeted therapy in human NHL is R-CHOP for B-cell NHL [110]. Although the addition of rituximab to CHOP chemotherapy has significantly improved the outcomes of patients with B-cell NHL, intrinsic or acquired resistance to rituximab can unfortunately be observed in some patients. Among the several proposed molecular mechanisms of resistance to rituximab, alterations of CD20 such as loss of expression on the cell surface [111,112,113] or mutations [114] are suggested to be associated with resistance. In veterinary medicine, the effects of several TKIs have been examined in canine mast cell tumors, and one study revealed that toceranib-resistant canine mastocytoma cell lines developed some mutations in the *c-kit* gene, which is the target of this drug [115]. However, molecular targeted therapy for canine lymphoma has not been established yet, and there was no report on the associations of drug target alterations with drug resistance in this disease.

### 5.2. Drug Detoxification

Glutathione S-transferase (GST) and glutathione (GSH) are the representative molecules associated with drug detoxification in drug-resistant tumor cells. Overexpression of these molecules promotes the detoxification of chemotherapeutic drugs such as alkylating agents and anthracycline and reduces the cytotoxic effects of these drugs [116]. In human chronic lymphocytic leukemia (CLL) cells, increased GST activity was suggested to be associated with chlorambucil resistance [117]. It was also suggested that the expression of GST-π had prognostic significance in human diffuse large B-cell lymphoma (DLBCL) patients [118]. We previously compared the expression level of the *GST-*α gene between canine lymphoma patients with and without drug-resistant phenotype; however, there was no significant difference [19]. Thus, the associations of GST or GSH with drug resistance are still unclear in veterinary medicine.

### 5.3. Increased DNA Damage Repair

Some of the commonly used chemotherapeutic drugs induce DNA damage to induce apoptosis in a proportion of tumor cells. Thus, a drug resistance phenotype can also come from the enhancement of the functions or the increase of the expressions of molecules involved in the process of DNA repair. O^6^-methylguanine-DNA methyltranserase (MGMT) is one of the molecules involved in DNA repair and interacts with the adducts on the O^6^-position of guanine [119]. As many chemotherapeutic agents, such as alkylating drugs, interact with DNA at this position to induce apoptosis, the increase in the expression of MGMT confers drug resistance phenotypes on the tumor cells. Especially, the associations of MGMT expressions or methylation status of the *MGMT* gene with the sensitivity to temozolomide have been well established in human glioma cells [120]. Also in human lymphoma, it was suggested that the detection of high expression of MGMT in immunohistochemistry might be associated with resistance to temozolomide in primary central nervous system lymphoma patients [121]. Although we previously examined the expression level of the *MGMT* gene in canine lymphoma, there was no significant difference between patients with and without drug resistance [19]. Thus, the associations of MGMT expression with drug resistance in tumor cells remain to be clarified in veterinary medicine.

### 5.4. Alterations in Apoptosis Pathways

As many chemotherapeutic agents exert antitumor effects by inducing apoptosis in tumor cells, some alterations in apoptosis signaling pathways are associated with drug resistance. In human lymphoma cells, several aberrations in the molecules associated with apoptosis pathways have been shown to induce drug resistance. Deficient levels of apoptotic protease-activating factor-1 (Apaf-1), which is involved in caspase pathways, were shown to be involved in the mechanisms of drug resistance in human Burkitt lymphoma [122]. Abnormalities in the tumor suppressor gene, *p53*, were also shown to be associated with drug resistance and short progression-free survival in human NHL patients [123]. The overexpression of the antiapoptotic proteins Bcl-2 was also reported to be associated with high relapse rates, short disease-free intervals and shorter overall survivals in human NHL [123,124,125,126,127]. In veterinary medicine, we previously compared the expression levels and the rates of mutations of the *p53* gene between drug-sensitive and -resistant canine lymphomas, but there was no significant difference [19]. However, another previous study showed that the positivity of P53 expression in immunohistochemistry significantly segregated the prognosis of canine patients with lymphoma [128]. Further studies are needed to reveal the role of these molecules in drug resistance in canine lymphoma.

## 6. Conclusions

In this review, we summarized recent studies on the molecular mechanisms of drug resistance in canine lymphoma in comparison with human lymphoma by focusing on the overexpression of drug transporters. Although the associations of drug resistance phenotypes with many molecules described here still remain to be clarified in veterinary medicine, it has been indicated that the overexpression of P-gp is one of the major causes of drug resistance in at least some canine lymphoma cases. Recent studies suggested some strategies such as the modulation of intracellular signaling pathways to overcome drug resistance in this disease. Based on these observations, tumor cell-specific interventions to modulate P-gp expression need to be investigated, and further studies are needed to develop these strategies *in vivo*. However, it is also known in human medicine that the expression of P-gp is not always linked to drug resistance *in vivo* and that the inhibition of the function of P-gp cannot always re-establish the sensitivity for chemotherapeutic drugs. These findings indicate that the molecular mechanisms of drug resistance are multifactorial and novel molecules should be identified to elucidate these mechanisms. Furthermore, it is possible that immunotherapy for canine lymphoma such as the therapy using the anti-CD20 antibody might be utilized in the near future. Therefore, alterations in the molecular targets of such therapies might become a new mechanism to overcome drug resistance as explored in human medicine.
